# The Uncertainties of Therapeutics

**Published:** 1906-04-21

**Authors:** 


### The Hospital

A Journal of

The Medical Sciences and Hospital Administration.

Vol. XL.—No. 1,021. SATURDAY, APRIL 21, 1906.

### The Uncertainties of Therapeutics.

The conflict of opinion regarding the actions and therapeutic values of many medicinal agents has been a painfully conspicuous fact throughout the entire history of the healing art. The pages of the past are crowded with such controversies, and in instances not a few the verdict of experience has condemned to utter neglect materials which at one time or another enjoyed high and widespread professional sanction. Nor can it be said that the more scientific methods of to-day have even yet established complete harmony. The quarrels and differences of our modern age are doubtless free from many of the extravagances and personal rancours of former times, but it still remains true that, whilst a more general agreement and a milder tone may both be recognised, there is still anything but a unanimous voice in the world of therapeutics. For past and present differences alike several causes must be held responsible. First among these must be placed imperfect and biassed observation. It is so easy to remember positive results which support a favourite theory, and to forget the facts which tell in the opposite direction that the judgment readily becomes warped unless the severe discipline of logical and scientific method is sedulously followed. It is to neglect of this training and influence that are to be traced many of the therapeutic claims which are by a longer experience shown to have no real and substantial foundation. Next may be mentioned a forgetfulness of the natural tendency of many of the processes of disease to decline or to terminate, with a corresponding return of the patient to his usual condition of health. Hence it follows that remedies used during the period of disturbance are apt to get the credit for a movement to which they did not really contribute, and which, as a matter of fact, has a natural process occurring altogether independently of the resources of art. Allied to this habit of imperfect reasoning is the impression made by the unexpected improvement or recovery of a patient from a more or less desperate position and the almost inevitable suggestion that the remedies in use at the time deserve credit for the change. Upon such scanty evidence the unscientific mind readily frames a large conclusion, and this not infrequently finds its expression through recognised channels, and may sooner or later receive endorsement from the text-books. All these factors contribute some share to the promotion of therapeutic confusion, and though their influence may be diminished by the cultivation of a more scientific habit of mind among members of the profession, it is hardly to be expected that, human nature being what it is, their force will ever entirely disappear. In addition, however, to factors which depend largely upon imperfectly disciplined mental habits, there are other circumstances which oppose therapeutic certainty. These arise from the urgency and complexity of the problem presented by the very existence of disease in an individual patient. Here, and more especially in an acute or dangerous illness, the conditions are not such as to admit the scientific methods appropriate to the laboratory. The aim to which the physician is compelled is not to discover or establish the value of any individual remedy, nor to decide the relative merits of a number of competing remedies. His main and direct purpose is neither to offer a contribution to pure pharmacological science nor to produce a therapeutic generalisation which shall be of benefit to mankind generally. On the contrary, his prime, even his sole, concern is with the individual patient, and the urgency of the position excludes, or at least subordinates, all other interests. Hence he is practically compelled to put into operation at one and the same time a number of agents which are more or less generally recognised as of value in the particular circumstances which he is called to meet; thus when success is attained he remains uncertain of the respective shares which these have contributed to the result. To proceed by the method of exclusion in individual cases with a view to gain an answer to this problem would be alien to the first and most sacred duty of the physician, which is, admittedly, to promote the welfare of his immediate patient. Thus, in the very nature of the case, there are elements in medical practice which exclude a precise and clean-cut therapeutic dogmatism, and it is in the interests of successful and efficient practice that this position should be realised. Lastly may be mentioned one of the consequences of the study of comparative pharmacology. No one questions that from this good results have accrued, and that much is yet to be expected. But it must at the same time be admitted that some laboratory results have come into conflict with certain well recognised methods of treatment, and that in this way there has been suggested a habit of therapeutic unrest and even of scepticism. That pharmacology may yet do much for therapeutics we have no doubt, but it must be remembered that the last word in the treatment of disease must be spoken by the practical physician, and it is on his careful observations and logical conclusions that such certainty as is possible in the circumstances must ultimately rest.

